# Long-term safety profile of sutimlimab in adult Japanese patients with cold agglutinin disease

**DOI:** 10.1007/s12185-024-03842-9

**Published:** 2024-10-14

**Authors:** Yoshitaka Miyakawa, Eriko Sato, Yoshiaki Ogawa, Jun-ichi Nishimura, Masashi Nishimi, Osamu Kawaguchi, Sayaka Tahara, Masaki Yamaguchi

**Affiliations:** 1 Department of Hematology, Saitama Medical University Hospital, 38 Morohongo Moroyama-machi, Iruma-gun, Saitama, 350-0495 Japan; 2 Division of Hematology, Department of Medicine, Juntendo University Nerima Hospital, Tokyo, Japan; 3 Department of Hematology/Oncology, Tokai University School of Medicine, Isehara, Japan; 4 Department of Hematology and Oncology, Osaka University Graduate School of Medicine, Osaka, Japan; 5 Sanofi K.K, Tokyo, Japan; 6 Department of Hematology, Ishikawa Prefectural Central Hospital, Kanazawa, Japan

**Keywords:** Cold agglutinin disease, Sutimlimab, Hemolytic anemia, Complement inhibitor, Long-term

## Abstract

Sutimlimab, a complement inhibitor, has recently been approved in Japan for treating cold agglutinin disease (CAD). We report the safety and efficacy of sutimlimab in Japanese patients with CAD who completed a global phase 3 clinical trial (CARDINAL/CADENZA: 26-week treatment with 1–2 years of open-label extension [OLE] periods) and subsequently participated in the Japanese OLE study. Patients with a recent history of blood transfusion (CARDINAL, n = 3) and those without (CADENZA, n = 4) were analyzed (71.4% female; median [range] baseline age: 70 [46–83] years). For CARDINAL/CADENZA, the treatment duration (median [range]) was 140.9 (104.9–157.3) weeks, and the cessation period was 70 (61–133) weeks. For the Japanese OLE study, the treatment duration was 47.1 (15.1–49.1) weeks. Three (42.9%) patients experienced treatment-related and treatment-emergent adverse events (TEAEs): injection site erythema, cystitis bacterial, viral infection, and blood pressure increased during CARDINAL/CADENZA. One (14.3%) patient experienced one treatment-related TEAE (urinary tract infection) during the Japanese OLE study. One patient died of renal failure, considered unrelated to sutimlimab, that was exacerbated by hepatorenal syndrome due to liver cirrhosis and bacterial peritonitis, in addition to CKD. Hemoglobin and bilirubin levels improved during treatment but deteriorated after withdrawal and recovered on retreatment. Sutimlimab was well tolerated over a median of 3.8 years, with no new safety concerns identified during retreatment.

**Supplementary Information:**

The online version contains supplementary material available at 10.1007/s12185-024-03842-9.

## Introduction

Cold agglutinin disease (CAD) is a chronic rare type of autoimmune hemolytic anemia [1–3] and is characterized by a clonal lymphoproliferative disorder that leads to production of monoclonal autoantibodies, including mainly immunoglobulin M with kappa light chain restriction (IgM-ĸ) [3–5]. These antibodies have the most effective binding affinity to the I surface antigen on erythrocytes at 0–4 °C, leading to erythrocyte agglutination [6, 7]. The agglutination causes symptoms such as acrocyanosis and Raynaud’s phenomenon [7]. Hemolysis in CAD is entirely protein complex C1 (complement component 1)-dependent, mediated by classical complement activation pathway [6].

The incidence of CAD varies across countries that have different climates [2, 8]. For instance, the crude incidence rates of CAD in Norway and Denmark are 1.8–1.9 cases per million per year, approximately 4 times higher than in Italy (0.5 cases per million per year) [9]. While a few reports suggest that the symptoms of CAD may be affected by seasonal changes and worsened in winter [10, 11], a large database study indicates evidence that chronic hemolysis persists year-round regardless of season in patients with CAD [12]. Patients with CAD experience chronic, ongoing classical complement pathway-mediated hemolysis interspersed with episodic hemolytic flares, resulting in chronic anemia and fatigue, affecting their quality of life [13]. CAD differs from cold agglutinin syndrome, which is transient and secondarily caused by infections, malignancies, or autoimmune disorders [14].

Sutimlimab is a first-in-class, humanized immunoglobulin G4 (IgG4) monoclonal antibody that specifically targets C1s. The mode of action of sutimlimab (TNT003) was confirmed through an in vitro study, which showed that TNT003 binds C1s at upstream of C4 and C2, thereby inhibiting cold agglutinin-mediated hemolysis [15]. This was further confirmed in a first-in-human study that sutimlimab inhibited complement pathway activities [16]. The results of global phase 3 clinical trials, CARDINAL and CADENZA, have demonstrated robust data that sutimlimab is effective and well tolerated in patients with CAD [17, 18]. Currently, sutimlimab is the only approved treatment in patients with CAD. Sutimlimab is a noncytotoxic pharmacotherapy that has attracted attention due to its mechanism of action as a classic pathway inhibitor with fast onset of action as well as high response rates and a favorable safety and tolerability profile [18, 19].

CARDINAL was an open-label, single-arm trial that enrolled 24 patients with CAD and a recent history (within the past 6 months) of blood transfusion [17], whereas CADENZA was a randomized, placebo-controlled trial that enrolled 42 patients with CAD without a recent history (within the past 6 months or > 1 within 12 months) of blood transfusion [18]. In both studies, sutimlimab showed sustained effects such as increased hemoglobin levels, reduced total bilirubin and lactate dehydrogenase (LDH) levels, as well as reduced fatigue, with only mild-to-moderate adverse events (AEs) as being related to sutimlimab, during the 26-week treatment period. Based on the results of these two studies, sutimlimab was approved in the USA in February 2022 [19], followed by Japan in June 2022 [20] and Europe in November 2022 [21] for the treatment of hemolysis in adult patients with CAD. The recently published results of the CARDINAL Part B (2-year extension) study demonstrated the sustained effects of sutimlimab on hemolytic and anemic markers and an acceptable safety profile that two (10%) of the patients experienced serious treatment-emergent AEs (TEAEs) being related to sutimlimab (vitreous hemorrhage and viral infection were experienced by one patient each) [22]. During the 2-year extension study of the CARDINAL trial, sutimlimab showed sustained effects on the disease activities throughout the year [22].

Given that CAD is a rare disease and sutimlimab was approved recently, the clinical data are still limited. Previous studies have indicated a recurrence of CAD symptoms after discontinuation of sutimlimab treatment, which was likely due to reversed complement inhibition [22]. Therefore, prolonged or lifelong use of sutimlimab may be desirable in patients with CAD. However, to date, safety and efficacy data for sutimlimab are only available for up to 2 years.

There are two aims to the open-label extension study from Japan (Japan OLE study): First, to provide continuous treatment to patients with CAD before obtaining regulatory approval. Second, to evaluate the long-term safety of sutimlimab by allowing periodic assessments of its use, including relatively extended withdrawal (cessation) periods (2–3 months), until the product became commercially available. We herein report the long-term safety and efficacy of sutimlimab in Japanese patients with CAD who completed the phase 3 CARDINAL/CADENZA study and continued in the Japan OLE study.

## Materials and methods

### Study design

**Fig. 1:**
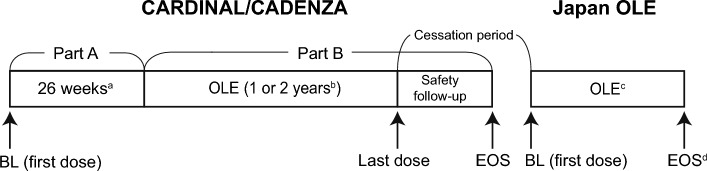
Study design. **a** Of the seven patients who participated in the Japan OLE study, one patient was assigned to placebo group in the CADENZA study during the 26-week treatment period (Part A). The study drug was administered on Day 0, Day 7, and subsequently every 2 weeks. **b** OLE phase (Part B) was 2 years in the CARDINAL study and 1 year in the CADENZA study after the last patient completed Part A, followed by a 9-week safety follow-up period. All patients received sutimlimab every 2 weeks. **c** Patients to receive sutimlimab until it is commercially available or discontinuation of study drug in the Japan OLE study. **d** The data were collected up to 2 weeks after the last (available) dose. *BL* baseline, *EOS* end of study, *OLE* open-label extension

The full study designs of the CARDINAL (NCT03347396) and CADENZA (NCT03347422) trials have been described previously [17, 18] (Fig. 1). Briefly, CARDINAL was a prospective, single-arm, open-label trial in patients with confirmed CAD and a recent history of blood transfusion within 6 months before enrollment. CADENZA was a placebo-controlled, randomized double-blind trial in patients with CAD, and the patients were excluded if they had a history of blood transfusion within 6 months (or a history of > ﻿1 blood transfusion within 12 months), before enrollment. Both studies consisted of a 26-week treatment period (Part A) followed by an open-label extension period (Part B). The duration of Part B was 2 years in the CARDINAL study and 1 year in the CADENZA study. The observation period included a 9-week follow-up period after the completion of Part B by the last patient.

The multicenter Japan OLE was a single-arm study with repeated dosing design conducted in Japanese patients with CAD who completed the Part B of the CARDINAL or CADENZA trial. A patient was considered to have completed the study if he/she had completed all phases of the study including the last visit.

The end of study was defined as 2 weeks after the last dose after the commercial availability of sutimlimab in Japan or at the end of the 9-week safety follow-up period, whichever occurred first. In cases where a patient was unable to switch to the commercial drug due to discontinuation of the study, they would proceed into a 9-week safety follow-up period.

### Intervention

In the Japan OLE study, the dosing regimen was the same as the CARDINAL and CADENZA trials: 6.5 g of sutimlimab for patients weighing ≥ 39 kg to < 75 kg, or 7.5 g for those weighing ≥ 75 kg at baseline. Sutimlimab was administered intravenously on Day 0, Day 7, and subsequently every 2 weeks. The data were collected up to 2 weeks following the last dose.

### Ethics

The study was performed in accordance with the Declaration of Helsinki, the International Conference on Harmonization Harmonized Tripartite Guideline for Good Clinical Practice, and all applicable local regulations. The study protocols were approved by institutional review boards of the study sites and written informed consent was obtained from all patients prior to their inclusion in the studies.

### Patient population

Key inclusion criteria in the CARDINAL/CADENZA trial included patients with hemoglobin levels ≤ 10.0 g/dL, total bilirubin level above normal range at screening and at least one CAD-related symptom within 3 months before screening [17, 18].

The Japan OLE study enrolled adult patients from Japan (aged ≥ 18 years, weighing ≥ 39 kg) with CAD who had completed Part B of the CARDINAL or CADENZA trial and who showed favorable efficacy and acceptable benefit/risk profiles during sutimlimab treatment. Moreover, those patients required continuous treatment for CAD, i.e., who showed relapse of CAD-related symptoms of anemia and/or deterioration on markers of hemolysis during the 9-week follow-up period.

In the Japan OLE study, patients were required to have received vaccination for *Neisseria meningitidis* and *Streptococcus pneumoniae* within 5 years before screening, and those who had experienced a serious infection with encapsulated bacteria within the past 3 months were excluded. Other exclusion criteria were patients who received rituximab monotherapy or similar immunosuppressive monotherapies within 3 months before screening or rituximab combination chemotherapies within 6 months before screening.

### Safety assessments

The safety assessments included AEs, clinical safety laboratory assessments (hematology, clinical chemistry, and urinalysis), vital signs, physical examinations, and pregnancy testing. Serious adverse events (SAEs) and adverse events of specific interest (AESIs; pregnancy and symptomatic overdose, etc.) were also assessed. AEs were coded according to the Medical Dictionary for Regulatory Activities (CARDINAL Part A, version 21.0; CARDINAL Part B, version 24.0; CADENZA Part A, version 23.0; CADENZA Part B, 24.1; Japan OLE, version 25.1). The exposure to sutimlimab for the safety population was measured by the duration of study intervention and total actual sutimlimab dose.

### Efficacy assessments

The Japan OLE study included patients who showed benefits from sutimlimab treatment in the CARDINAL or CADENZA study; hence, the assessments of efficacy were not predefined. However, with the aim of collecting clinical data, levels of hemoglobin, bilirubin and LDH were collected at both baseline and the end of the study (i.e., 2 weeks after the last dose of sutimlimab), and these were reported as efficacy outcomes.

### Statistical analysis

No power and sample size analyses were conducted. The primary analyses were performed on the safety population defined as all patients who received at least one dose of sutimlimab. Safety data were summarized using descriptive statistics for continuous and categorical data.

## Results

The studies were conducted from March 2018 to October 2021 in the CARDINAL trial, from March 2018 to November 2021 in the CADENZA trial, and from November 2021 to November 2022 in the Japan OLE trial. The total median (range) observation period of this report (i.e., from the enrollment of CARDINAL/CADENZA to the end of the Japan OLE study) was 3.8 (3.2–4.2) years.

### Patient disposition and baseline characteristics

Seven Japanese patients completed the Part B of the CARDINAL/CADENZA study from five Japanese investigational sites (CARDINAL, n = 3; CADENZA, n = 4 [sutimlimab, n = 3; placebo, n = 1 in Part A]), and all entered the Japan OLE study. All of them received at least one dose of sutimlimab and were in the 6.5 g dose cohort. The median (range) age of patients at baseline in the CARDINAL/CADENZA studies was 70 (46–83) years and the majority were female (n = 5, 71.4%) (Table 1). One (14.3%) patient discontinued the study drug due to treatment-emergent serious AEs (TESAEs) associated with worsening of chronic kidney disease (CKD); thus, six patients completed the Japan OLE study.

**Table 1:** Patient disposition and baseline characteristics

	Median (range), N = 7
*CARDINAL/CADENZA*	
Age at the enrollment, years	70.0 (46–83)
Sutimlimab treatment duration, weeks	140.9 (104.9–157.3)
Total sutimlimab dose, gram	461.5 (344.5–513.5)
Baseline hemoglobin level, g/dL	8.7 (6.2–12.7)
Baseline serum bilirubin level, mg/dL	2.0 (1.5–2.6)
Baseline serum LDH level, U/L	304.0 (160–452)
*Japan OLE*	
Sutimlimab treatment duration, weeks	47.1 (15.1–49.1)
Total sutimlimab dose, gram	156.0 (52.0–162.5)
Baseline hemoglobin level, g/dL	8.8 (6.3–11.3)
Baseline serum bilirubin level, mg/dL	2.7 (1.4–4.6)
Baseline serum LDH level, U/L	348.0 (254–800)
*Cessation period*^a^, *days*	
Overall (N = 7)	70 (61–133)
CARDINAL completer (n = 3)	127 (126–133)
CADENZA completer (n = 4)	65 (61–70)

^a^Cessation period is defined as the period between the last dose of the CARDINAL/CADENZA study and the first dose of the Japan OLE study

*LDH* lactate dehydrogenase, *OLE *open-label extension

In the CARDINAL/CADENZA study, the median (range) duration of study treatment was 140.9 (104.9–157.3) weeks with the actual median (range) study dose of 461.5 (344.5−513.5) g. The median (range) cessation period between the last dose in the CARDINAL/CADENZA and the first dose in the Japan OLE study was 70 (61–133) days. The median (range) cessation period was longer in the patients who participated in CARDINAL study than those who participated in CADENZA study, 127 (126–133) vs 65 (61–70) days, respectively. In the Japan OLE study, the median (range) duration of sutimlimab treatment was 47.1 (15.1–49.1) weeks with an actual median (range) study dose of 156.0 (52.0−162.5) g (Table 1). No patients completed the 9-week safety follow-up period of the Japan OLE study, six patients switched to commercially available sutimlimab, and one patient died due to chronic kidney disease during the safety follow-up period (26 days after the last dose of sutimlimab) following discontinuation of the study drug. Compliance with the planned dosing schedule was 100%.

All four patients who transitioned from CADENZA to the Japan OLE study were vaccinated against COVID-19 during CADENZA Part B. Two of the three patients who transitioned from CARDINAL to the Japan OLE study were vaccinated during CARDINAL Part B. Overall, five of seven patients received COVID-19 vaccination during the Japan OLE study, with three receiving elasomeran and three receiving tozinameran.

### Safety

During the CARDINAL/CADENZA study, all seven (100%) patients experienced at least one TEAE, with a total of 106 TEAEs. Of these, the most frequently (> 2 patients) reported TEAEs were iron deficiency anemia, constipation, and nasopharyngitis, experienced by three patients each. Injection site erythema, cystitis bacterial, viral infection, and blood pressure increased were assessed as treatment-related TEAEs and experienced by a total of three patients (42.9%) (Table 2A). Three (42.9%) patients reported at least one TESAE with hemorrhoids, viral infection and Raynaud’s phenomenon experienced by one patient each, where viral infection was assessed as related to study drug by the investigator.

**Table 2:** Safety summaries during (**A**) the CARDINAL/CADENZA studies and (**B**) the Japan OLE study

**A **	Total (N = 7)
Total number of TEAEs, n	106
Patients with at least one TEAE, n (%)	7 (100)
Most frequently reported TEAEs (≥ 3 patients), n (%)	
Iron deficiency anemia	3 (42.9)
Constipation	3 (42.9)
Nasopharyngitis	3 (42.9)
Patients with at least one related TEAE, n (%)	3 (42.9)
Injection site erythema	1 (14.3)
Cystitis bacterial	1 (14.3)
Viral infection	1 (14.3)
Blood pressure increased	1 (14.3)
Total number of TESAEs, n	3
Patients with at least one TESAE, n (%)	3 (42.9)
Hemorrhoids	1 (14.3)
Viral infection	1 (14.3)
Raynaud’s phenomenon	1 (14.3)
Patients with at least one related TESAE, n (%)	1 (14.3)
Viral infection	1 (14.3)

**B**	Total (N = 7)
Total number of TEAEs, n	46
Patients with at least one TEAE, n (%)	7 (100)
Most frequently reported TEAEs (≥ 2 patient), n (%)	
Back pain	3 (42.9)
Pyrexia	2 (28.6)
Patients with at least one related TEAE, n (%)	1 (14.3)
Urinary tract infection	1 (14.3)
Total number of TESAEs, n	3
Patients with at least one TESAE, n (%)	1 (14.3)
Cholangitis acute	1 (14.3)
Spontaneous bacterial peritonitis	1 (14.3)
Chronic kidney disease	1 (14.3)
Patients with at least one related TESAE, n (%)	0
Patients who discontinued treatment and/or study due to a TEAE, n (%)	1 (14.3)
Deaths, n (%)	1 (14.3)^a^

Patients were counted once if they reported multiple events in the same preferred term

Percentages are based on the number of patients enrolled in the Japan OLE study

AEs with missing causality assessment are included in the related TEAE or related TESAE

^a^One patient died of chronic kidney disease (unrelated to the study treatment)

*AE﻿* adverse event, *OLE* open-label extension, *TEAE* treatment-emergent adverse event, *TESAE* treatment-emergent serious adverse event

In the Japan OLE study, all seven (100%) patients experienced at least one TEAE, with a total of 46 TEAEs. One (14.3%) patient had at least one TEAE (urinary tract infection) assessed as related to sutimlimab by the investigator. The event was nonserious and mild in severity and resolved after medication. The most frequently (≥ 20% [i.e., ≥ 2 patients]) reported TEAEs (by preferred term) were back pain (3; 42.9%) and pyrexia (2; 28.6%).

In the Japan OLE study, TESAEs and TEAEs leading to treatment discontinuation were reported in the same patient aged 81 years old who died during the study (Table 2B). This patient had chronic kidney disease (CKD), renal anemia, liver damage, and fatty liver at the time of enrollment in the Japan OLE study. During the safety follow-up period, 26 days after the last dose of sutimlimab, the patient died of renal failure exacerbated by hepatorenal syndrome due to liver cirrhosis, bacterial peritonitis in addition to CKD. This patient experienced three TESAEs which included: cholangitis acute on Day 17, spontaneous bacterial peritonitis on Day 81, and exacerbated CKD on Day 83. Two of these three TESAEs (spontaneous bacterial peritonitis and CKD) led to discontinuation of the study intervention and/or the study. The investigator did not consider any of these TESAEs as related to sutimlimab; cholangitis acute was attributed to gallstones, spontaneous bacterial peritonitis to transudative ascites in association with hepatic cirrhosis, and CKD to the underlying medical condition being exacerbated by transudative ascites, in association with hepatic cirrhosis, and spontaneous bacterial peritonitis, resulting in continuous hemodiafiltration.

No AESIs were reported throughout the observation period.

### Efficacy

**Fig. 2:**
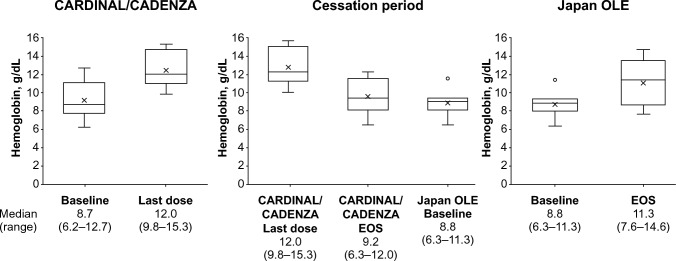
Box–whisker plot for serum hemoglobin levels at each time point. The upper and lower limits of the box plot denote interquartile ranges; whiskers denote maximum and minimum ranges. The line and X in the box plot denote median and mean, respectively. The circle denotes outlier. The number below each timepoint denotes median (range) of the hemoglobin levels (N = 7). EOS, end of study; OLE, open-label extension

In the analysis population (n = 7), the median (range) hemoglobin level increased from 8.7 (6.2–12.7) g/dL at baseline to 12.0 (9.8–15.3) g/dL at the last dose of sutimlimab in the CARDINAL/CADENZA study (Fig. 2, Supplementary Fig. 1). The hemoglobin level decreased during the cessation period to 8.8 (6.3–11.3) g/dL at the baseline of the Japan OLE study; however, hemoglobin levels increased to 11.3 (7.6–14.6) g/dL at the end of the study.

**Fig. 3:**
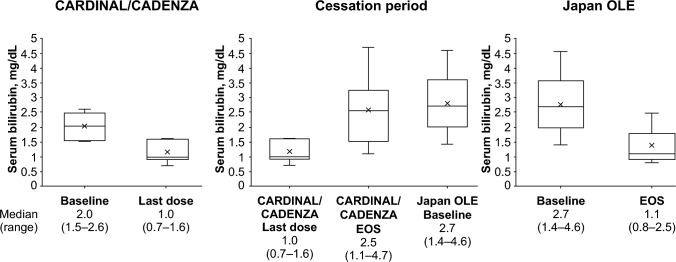
Box–whisker plot for serum bilirubin levels at each time point. The upper and lower limits of the box plot denote inter-quartile ranges, whiskers denote maximum and minimum ranges. The line and X in the box plot denote median and mean, respectively. The number below each timepoint denotes median (range) of the bilirubin levels (N = 7). EOS, end of study; OLE, open-label extension

In the CARDINAL/CADENZA study, the median (range) total bilirubin level decreased from 2.0 (1.5–2.6) mg/dL at baseline to 1.0 (0.7–1.6) mg/dL at the last dose of sutimlimab (Fig. 3, Supplementary Fig. 2). The total bilirubin increased to 2.7 (1.4–4.6) mg/dL during the cessation period of the CARDINAL/CADENZA study to the baseline of the Japan OLE study; however, total bilirubin decreased again to 1.1 (0.8–2.5) mg/dL at the end of the Japan OLE study.

In both studies, LDH levels varied widely among patients. In the CARDINAL/CADENZA study, the median (range) LDH level decreased from 304.0 (160.0–452.0) U/L at baseline to 266.0 (190.0–651.0) U/L at the last dose of sutimlimab in the CARDINAL/CADENZA study. The LDH level increased during the cessation period to 348.0 (254.0–800.0) U/L at the baseline of the Japan OLE study and increased to 534.0 (168.0–1284.0) U/L at the end of the study. Although the median LDH level increased during the Japan OLE study period, three out of seven patients showed a decrease in LDH levels from baseline in the Japan OLE study (Supplementary Figs. 3 and 4).

In general, all patients exhibited deterioration in the anemic and hemolytic markers after discontinuation of sutimlimab: decrease in hemoglobin level, increase in bilirubin level during the cessation period. However, these levels were restored during the Japan OLE study. The LDH level varied individually, and no improvement was observed in the median LDH level during the Japan OLE study.

One patient who participated in the CADENZA study and was assigned to the sutimlimab treatment group received blood transfusion during Part A (26-week treatment period). Two patients received blood transfusion during the cessation period (i.e., between the last dose of the CARDINAL/CADENZA study and the first dose of the Japan OLE study); however, none of the patients required blood transfusion after sutimlimab administration during the Japan OLE study.

## Discussion

The Japan OLE study was a multicenter, single-arm, open-label, phase 3 extension study designed to evaluate the long-term safety profile (median, 3.8 years) of sutimlimab in adult patients with CAD. This report includes the safety data of sutimlimab in Japanese patients combining both CARDINAL/CADENZA and Japan OLE studies. The Japan OLE study intended to provide continuous treatment for the patients who previously benefitted from sutimlimab through clinical trials until regulatory approval of sutimlimab was obtained in Japan. The entire observation period covered in this report included the period of drug cessation, as the Japan OLE study commenced after the 9-week safety follow-up (drug withdrawal) period of the CARDINAL/CADENZA study.

Overall, similar to the safety data of the CARDINAL/CADENZA study, sutimlimab was well tolerated in the Japan OLE study, where patients received retreatment of sutimlimab. There were no apparent trends in the reported TEAEs in both studies, which were generally nonserious, mild or moderate in severity and clinically manageable; three patients experienced treatment-related TEAEs, with one treatment-related TESAE during the CARDINAL/CADENZA study. During the Japan OLE study, one patient experienced one treatment-related TEAE. TESAEs and TEAEs leading to treatment discontinuation were reported in the same patient who died in the Japan OLE study. This patient had CKD, renal anemia, liver cirrhosis, and fatty liver at the time of enrollment of the Japan OLE study and died of renal failure, worsened by hepatorenal syndrome due to liver cirrhosis and bacterial peritonitis in addition to CKD. The three TESAEs (cholangitis acute, spontaneous bacterial peritonitis, and CKD) that were experienced by this patient were considered as not related to sutimlimab.

Sutimlimab is a selective inhibitor for C1s of the classical complement pathway. Theoretically, classical complement inhibitors are suspected to have a potential risk for severe infection caused by encapsuled bacteria, including *Neisseria meningitidis*, *Streptococcus pneumoniae,* and *Haemophilus influenzae type b* [1, 23]. Although patients were required to have received vaccinations against encapsulated organisms (*Neisseria meningitidis* and *Streptococcus pneumoniae*) in the sutimlimab phase 3 clinical trials, no such bacterial infection was reported during the CARDINAL/CADENZA or the Japan OLE study period. During the Japan OLE study, which coincided with the COVID-19 pandemic, investigators were recommended to vaccinate enrolled patients. In a case series of four patients, CAD exacerbations have been reported after both COVID-19 infection and vaccination [24]. Consistent with a previous post-hoc analysis, the results of the Japan OLE study showed no breakthrough hemolysis in five out of seven patients who received the COVID-19 vaccine [25]. This previous analysis investigated the safety and immunogenicity of vaccination against COVID-19 in patients receiving sutimlimab during the OLE Part B of the CARDINAL/CADENZA studies [25]. The findings revealed no occurrence of serious AEs, and there were no indications of hemolytic anemia or hemolytic flares/breakthrough hemolysis after vaccination. Comparable levels of hemoglobin, total bilirubin, and lactate dehydrogenase pre- and post-vaccination were reported. It also reported that the immunogenic response to one, two, or booster doses remained unaffected, with all patients exhibiting positive titers against the SARS-CoV-2 spike protein [25].

The findings of this analysis provide additional insights into the favorable safety profile of sutimlimab over a long-term treatment duration. The retreatment with sutimlimab following the cessation period was well tolerated, and no new safety signals were identified.

Favorable changes in anemic and hemolytic markers were observed during the retreatment period in the Japan OLE study, which were similar to those observed during the treatment period of the CARDINAL and CADENZA studies. This analysis included a median (range) of 70 (61–133) days from the cessation period, which were relatively long particularly for the three patients who participated in the CARDINAL study (median [range], 127 [126–133] days).

In this analysis, discontinuation of sutimlimab led to decrease in hemoglobin and increase in bilirubin levels, which is in line with previous reports [1, 26, 27]. However, those levels were restored in all patients during the Japan OLE study and no patients required blood transfusions. This result supports the premise that sutimlimab may be effective when used periodically. The median LDH level increased from baseline to the end of the Japan OLE study. This is likely because of the small sample size and high variation among the patients. For instance, the LDH level of one patient was prominently elevated at the end of the Japan OLE study. A similar trend (i.e., an elevated LDH level) was observed in this patient during the CADENZA study (Supplementary Fig. 3 and 4). During the CADENZA study, the change in LDH levels in this patient was likely to be affected by the seasonal temperature (i.e., increased in winter and decreased in summer [12]); thus, influence of the seasonal temperature cannot be precluded. However, measurement time points at baseline and at the end of the Japan OLE study were in the same month (November); therefore, the cause of an increase in the median LDH level observed in the Japan OLE study remains unclear. Serum LDH level may be a less sensitive marker of immune hemolytic anemia than of intravascular hemolysis because serum LDH does not significantly increase in extravascular hemolysis [28]. In any case, a further analysis with a larger sample size, more time points, and markers will be required to elucidate the mechanism of elevated LDH.

## Limitations

The Japan OLE study was conducted in Japan, and the analysis was conducted in patients who completed previous clinical trials from Japan; hence, the sample size was small. Therefore, the results may limit the generalizability of the data, and no statistical power analysis was applied.

Owing to the purpose and nature of the study, the efficacy analysis was limited. However, considering that CAD is a rare disease, any data of relatively few patients that are added to globally available data may provide insights into future clinical practice.

In conclusion, sutimlimab was generally well tolerated over a median 3.8 years of the long-term observation period including retreatment, and no new safety concerns were identified.

## Supplementary Information

Below is the link to the electronic supplementary material.

Supplementary file1 (PDF 2369 KB)

## Data Availability

Qualified researchers may request access to patient-level data and related study documents including the clinical study report, study protocol with any amendments, blank case report form, statistical analysis plan, and dataset specifications. Patient level data will be anonymized, and study documents will be redacted to protect the privacy of our trial participants. Further details on Sanofi’s data sharing criteria, eligible studies, and process for requesting access can be found at https://www.vivli.org/.
