# Supplementary material for: Long-term safety profile of sutimlimab in adult Japanese patients with cold agglutinin disease
Source: Int J Hematol. 2024 Oct 14;120(6):656–64. doi: 10.1007/s12185-024-03842-9 (PMC11588873; doi:10.1007/s12185-024-03842-9)
Supplement: Supplementary file 1 — Supplementary file1 (PDF 2369 KB) [file 12185_2024_3842_MOESM1_ESM.pdf]

Long-term safety of sutimlimab in adult Japanese patients with cold agglutinin disease

Yoshitaka Miyakawa[1], Eriko Sato[2], Yoshiaki Ogawa[3], Jun-ichi Nishimura[4], Masashi Nishimi[5], Osamu Kawaguchi[5], Sayaka Tahara[5], and Masaki Yamaguchi[6]

[1] Department of Hematology, Saitama Medical University Hospital, Saitama, Japan

[2] Division of Hematology, Department of Medicine, Juntendo University Nerima Hospital, Tokyo, Japan

[3] Department of Hematology/Oncology, Tokai University School of Medicine, Isehara, Japan

[4] Department of Hematology and Oncology, Osaka University Graduate School of Medicine, Osaka, Japan

[5] Sanofi K.K., Tokyo, Japan

[6] Department of Hematology, Ishikawa Prefectural Central Hospital, Kanazawa, Japan

Correspondence:
Yoshitaka Miyakawa, MD, PhD
Department of Hematology
Saitama Medical University Hospital
38 Morohongo Moroyama-machi, Iruma-gun, Saitama, 350-0495, Japan
Email: miyakawa@saitama-med.ac.jp

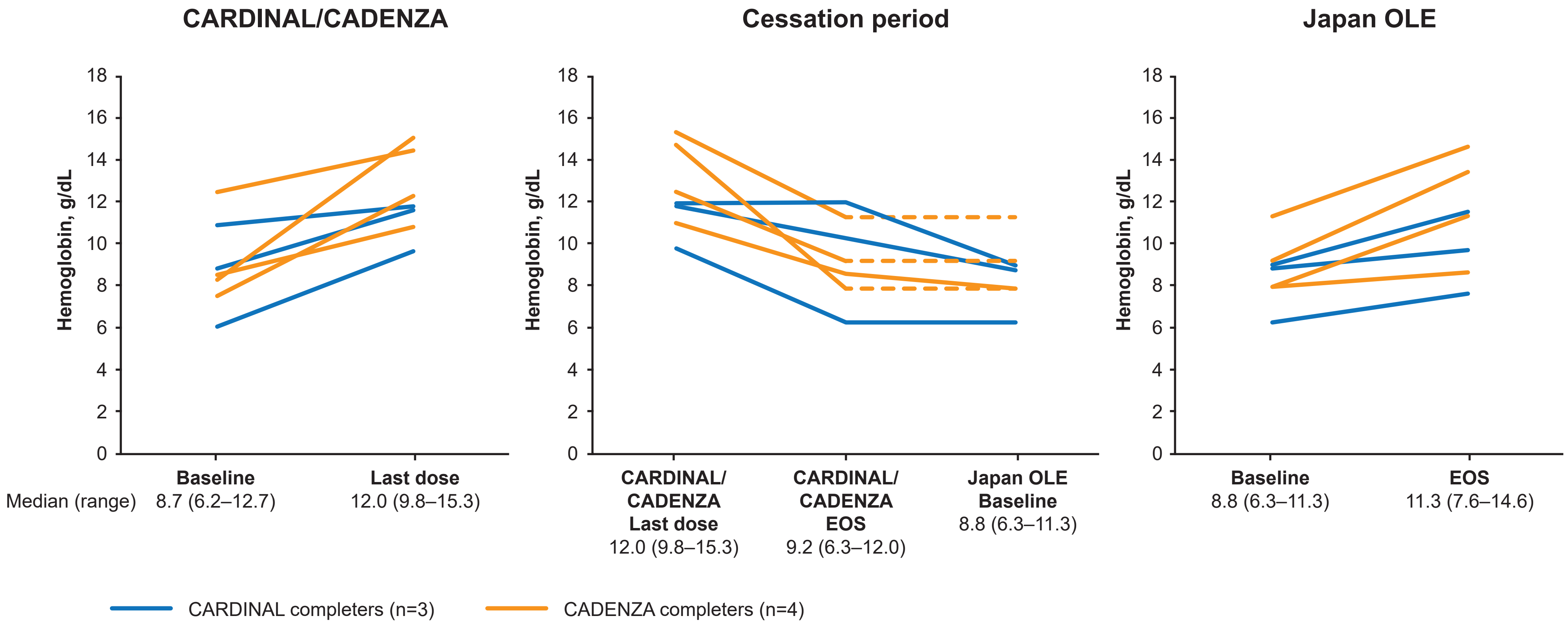

Supplementary Figure 1. Changes in hemoglobin levels in individual patients.
Three patients in the CADENZA entered Japan OLE study immediately after the end of CADENZA study (dotted orange line).
The number below each timepoint denotes median (range) of the hemoglobin levels (N=7).
EOS, end of study; OLE, open-label extension

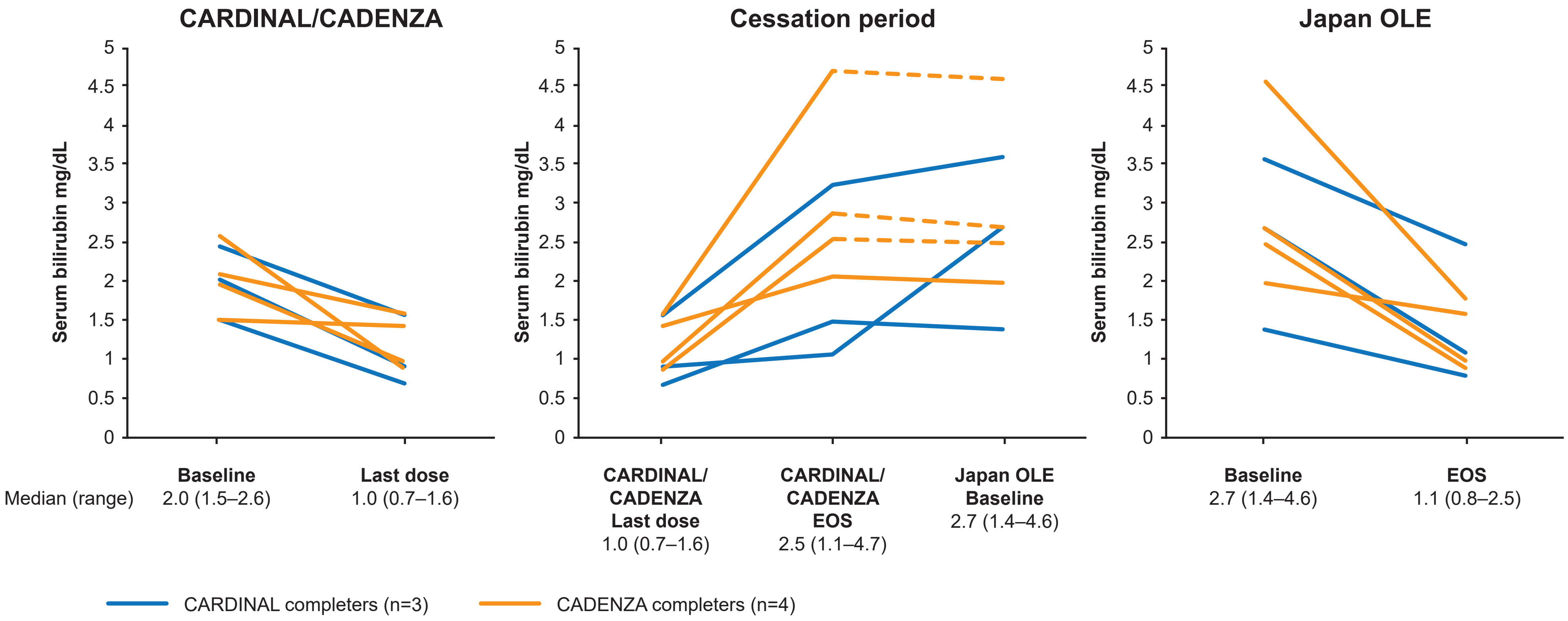

Supplementary Figure 2. Changes in bilirubin levels in individual patients.
Three patients in the CADENZA entered Japan OLE study immediately after the end of CADENZA study (dotted orange line).
The number below each timepoint denotes median (range) of the bilirubin levels (N=7).
EOS, end of study; OLE, open-label extension

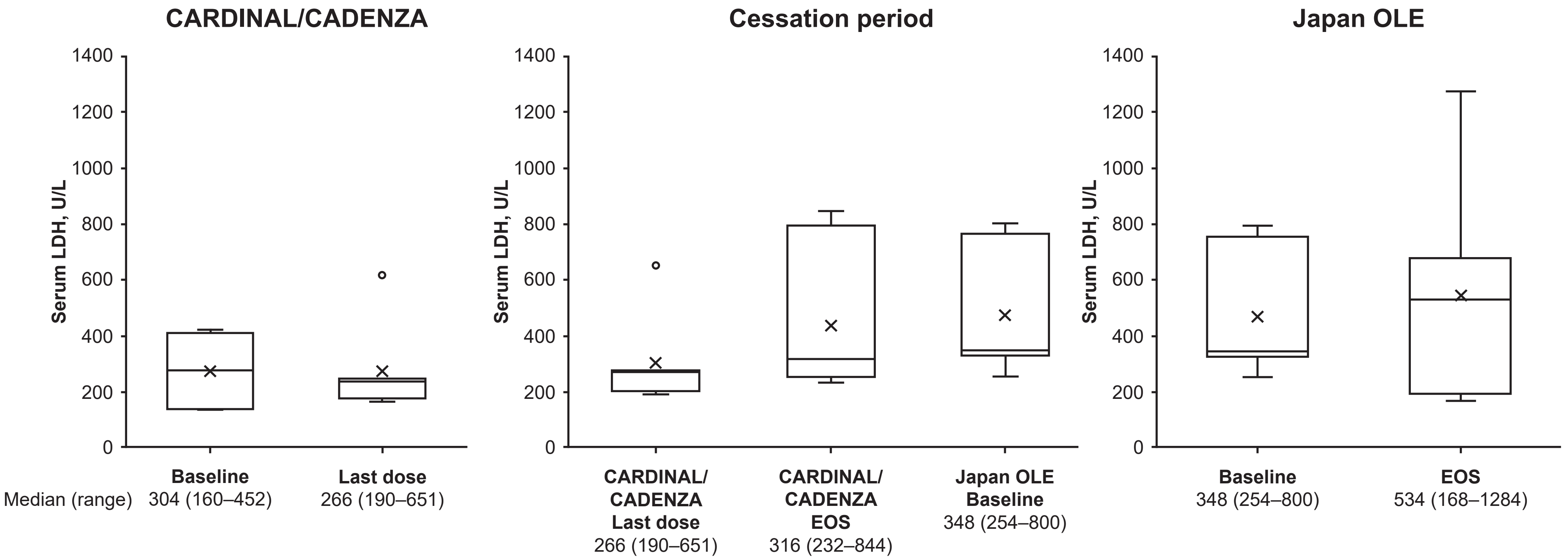

Supplementary Figure 3. Box-whisker plot for serum LDH levels at each time point.

The upper and lower limits of the box plot denote inter-quartile ranges, whiskers denote maximum and minimum ranges. The line and X in the box plot denote median and mean, respectively. The circle denotes outlier. The number below each timepoint denotes median (range) of the LDH levels (N=7).

EOS, end of study; LDH, lactate dehydrogenase; OLE, open-label extension

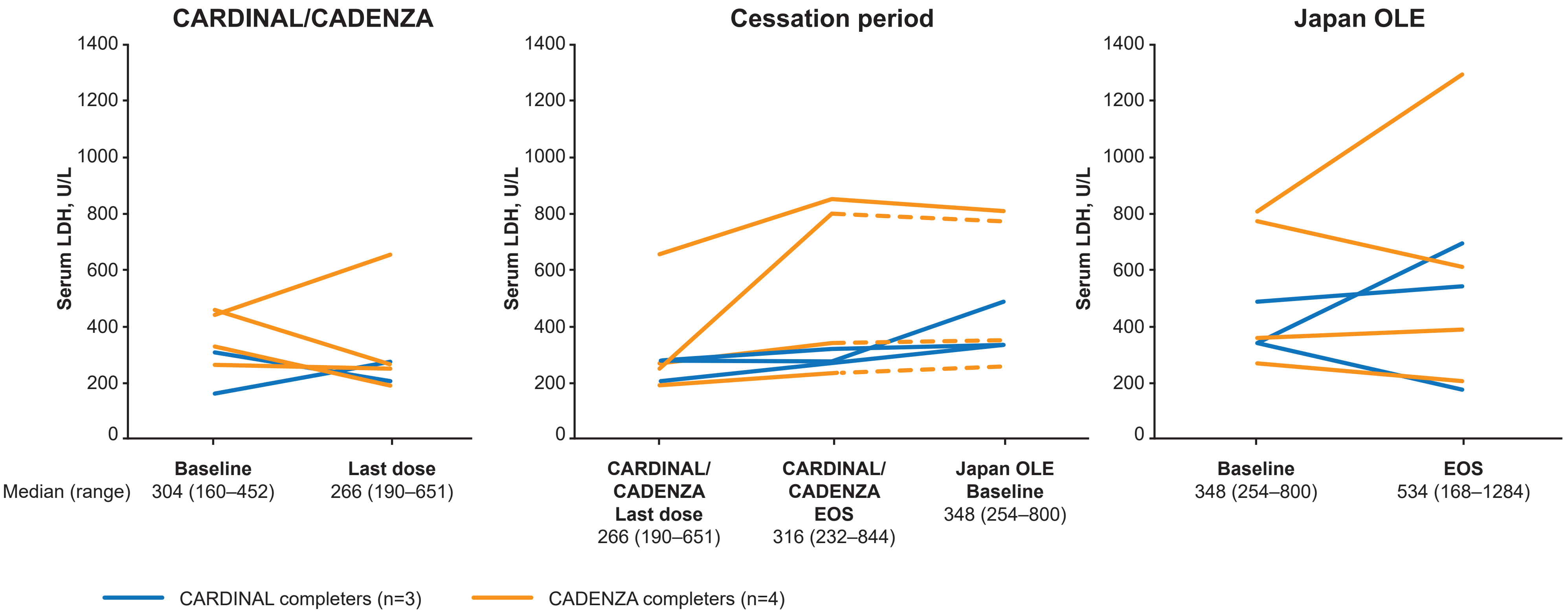

Supplementary Figure 4. Changes in LDH levels in individual patients.
Three patients in the CADENZA entered Japan OLE study immediately after the end of CADENZA study (dotted orange line).
The number below each timepoint denotes median (range) of the LDH levels (N=7).
EOS, end of study; LDH, lactate dehydrogenase; OLE, open-label extension
